# Supplementary material for: Physical well-being recovery trajectories by reconstruction modality in women undergoing mastectomy and breast reconstruction: Significant predictors and health-related quality of life outcomes
Source: PLoS One. 2023 Jul 28;18(7):e0289182. doi: 10.1371/journal.pone.0289182 (PMC10381031; doi:10.1371/journal.pone.0289182)
Supplement: S1 File — (DOCX) [file pone.0289182.s001.docx]

**S1 Table1. Definition of candidate variables and outcomes.**

| **Variable** | **Classification** | **Definition** |
| --- | --- | --- |
| **Patient variables** | | |
| Age | Numerical | Age reported by patients at baseline |
| BMI | Numerical | BMI as reported by the patient |
| Diabetes | Categorical | Diabetes if the patient had or not |
|  | (1)No |  |
|  | (2)Yes [reference] |  |
| Smoker | Categorical | Smoking status as reported by patients. “no” category includes “never” and “previous” smokers. |
|  | (1)No |  |
|  | (2)Yes [reference] |  |
| **Pre-operative patient-reported outcome data** | | |
| BREAST-Q satisfaction with breast | Numerical | Breast satisfaction score ranging from 0 to 100 reported by patients at baseline |
| BREAST-Q physical well-being chest and upper body | Numerical | Physical well-being score ranging from 0 to 100 reported by patients at baseline |
| BREAST-Q psychosocial well-being | Numerical | Psychosocial well-being score ranging from 0 to 100 reported by patients at baseline |
| BREAST-Q physical well-being abdomen | Numerical | Physical well-being abdomen score ranging from 0 to 100 reported by patients at baseline |
| BREAST-Q sexual well-being | Numerical | Sexual well-being score ranging from 0 to 100 reported by patients at baseline |
| Follow-up patient-reported outcome data | | |
| BREAST-Q physical well-being chest and upper body at 3 months | Numerical | Physical well-being score ranging from 0 to 100 reported by patients at 3-month follow up |
| BREAST-Q physical well-being chest and upper body at 12 months | Numerical | Physical well-being score ranging from 0 to 100 reported by patients at 1-year follow up |
| BREAST-Q physical well-being chest and upper body at 24 months | Numerical | Physical well-being score ranging from 0 to 100 reported by patients at 2-year follow up |
| BREAST-Q psychosocial well-being at 24 months | Numerical | Psychosocial well-being score ranging from 0 to 100 reported by patients at 2-year follow up |
| BREAST-Q sexual well-being at 24 months | Numerical | Sexual well-being score ranging from 0 to 100 reported by patients at 2-year follow up |
| BREAST-Q breast satisfaction at 24 months | Numerical | Breast satisfaction score ranging from 0 to 100 reported by patients at 2-year follow up |
| Radiation | Categorical | Radiation therapy received before or after reconstruction |
|  | (1)Before reconstruction |  |
|  | (2)None |  |
|  | (3)After reconstruction[reference] |  |
| Mastectomy | Categorical | Mastectomy type patients received |
|  | (1)Simple |  |
|  | (2)Nipple-sparing[reference] |  |
| Chemotherapy | Categorical | Chemotherapy received by patient during or after reconstruction |
|  | (1)Received |  |
|  | (2)Not received[reference] |  |
| Reconstruction laterality | Categorical | Reconstruction laterality type as reported by patients |
|  | (1)Unilateral |  |
|  | (2)Bilateral[reference] |  |
| Mastectomy indication | Categorical | Indication for mastectomy as reported by patients |
|  | (1)Therapeutic |  |
|  | (2)Prophylactic [reference] |  |
| Axillary intervention | Categorical | Axillary intervention type as reported by patients |
|  | (1)None |  |
|  | (2)Sentinel lymph node biopsy (SLNB) |  |
|  | (3)Axillary lymph node dissection (ALND) [reference] |  |
| **Socioeconomic and ethnic data** | | |
| Marital status | Categorical | Simplified marital status reported by patients |
|  | (1)Partnerless |  |
|  | (2)Partnered [reference] |  |
| Education level | Categorical | Simplified education level reported by patients |
|  | (1)High school degree and below |  |
|  | (2)Above high school degree[reference] |  |
| Working status | Categorical | Simplified working status reported by patients. “Other” category contains “unable to work”, “unemployed”, “student”, “volunteer”, “retired”, “homeworker” status. |
|  | (1)Others |  |
|  | (2)Employed [reference] |  |
| Household income per year | Categorical | Simplified annual household income reported by patients |
|  | (1) <$50,000 |  |
|  | (2) $50,000 to $99,999 |  |
|  | (3) >$100,000[reference] |  |
| Race | Categorical | Simplified race reported by patients |
|  | (1)White |  |
|  | (2)Non-White[reference] |  |
| **Health-related quality of life outcome** | | |
| Breast satisfaction change | Categorical | The changes of breast satisfaction equal or greater than minimal clinically important difference (MCID) of 4 defines the 3 outcome types of improved, worsened, stable breast satisfaction for patients |
|  | (1)Improved |  |
|  | (2)Worsened |  |
|  | (3)Stable [reference] |  |
| Psychosocial well-being change | Categorical | The changes of psychosocial well-being equal or greater than MCID of 4 defines the 3 outcome types of improved, worsened, stable psychosocial well-being for patients |
|  | (1)Improved |  |
|  | (2)Worsened |  |
|  | (3)Stable [reference] |  |
| Sexual well-being change | Categorical | The changes of sexual well-being equal or greater than MCID of 4 defines the 3 outcome types of improved, worsened, stable sexual well-being for patients |
|  | (1)Improved |  |
|  | (2)Worsened |  |
|  | (3)Stable [reference] |  |

**S1 Table 2. Predictive mean and sample mean of breast reconstruction techniques across time.**

|  |  |  | **Time (Month)** | | | |
| --- | --- | --- | --- | --- | --- | --- |
|  | **Recoded name** | **n** | **0** | **3** | **12** | **24** |
| **Implant-based reconstruction(n=843)** | | | | | | |
| Class 1 | medium high-not restored | 87 |  |  |  |  |
| Predictive mean |  |  | 89.77 | 64.86 | 65.13 | 70.22 |
| Sample mean |  |  | 94.53 | 64.36 | 62.77 | 67.62 |
| Class 2 | high-restored | 266 |  |  |  |  |
| Predictive mean |  |  | 90.47 | 79.47 | 88.63 | 90.47 |
| Sample mean |  |  | 91.16 | 79.73 | 89.12 | 91.11 |
| Class 3 | low-not restored | 110 |  |  |  |  |
| Predictive mean |  |  | 63.83 | 54.53 | 56.88 | 56.97 |
| Sample mean |  |  | 63.28 | 53.64 | 56.08 | 56.15 |
| Class 4 | medium low-restored | 380 |  |  |  |  |
| Predictive mean |  |  | 76.07 | 67.80 | 74.83 | 76.13 |
| Sample mean |  |  | 75.44 | 67.59 | 74.52 | 75.95 |
| **Autologous reconstruction(n=581)** | | | | | | |
| Class 1 | medium high-restored | 261 |  |  |  |  |
| Predictive mean |  |  | 79.01 | 74.87 | 77.71 | 79.71 |
| Sample mean |  |  | 79.20 | 75.25 | 78.04 | 79.82 |
| Class 2 | low-not restored | 28 |  |  |  |  |
| Predictive mean |  |  | 58.89 | 49.70 | 47.30 | 44.42 |
| Sample mean |  |  | 56.93 | 49.96 | 46.04 | 41.93 |
| Class 3 | medium low-not restored | 187 |  |  |  |  |
| Predictive mean |  |  | 67.80 | 62.38 | 64.47 | 64.41 |
| Sample mean |  |  | 67.17 | 61.43 | 63.72 | 63.50 |
| Class 4 | high-restored | 105 |  |  |  |  |
| Predictive mean |  |  | 91.25 | 85.61 | 92.82 | 91.25 |
| Sample mean |  |  | 92.90 | 86.39 | 94.31 | 93.14 |

**S1 Table 3. Baseline demographic and clinical characteristics of participants with implant-based reconstruction in distinct physical well-being trajectories (n=843).**

|  | **Class1** | **Class2** | **Class 3** | **Class 4** | ***p*-value^a^** |
| --- | --- | --- | --- | --- | --- |
|  | medium high-not restored  (n=87) | high-restored  (n= 266) | low-not restored  (n= 110) | medium low-restored  (n=380) |  |
| **Patient variables** | | | | | |
| Age, mean (SD), years | 48.00(9.40) | 48.56(10.60) | 48.95(9.82) | 48.56(10.47) | 0.938^b^ |
| BMI, mean (SD), kg/m^2^ | 25.04(5.05) | 24.23(4.21) | 26.78(5.81) | 24.93(4.90) | **<0.001^b^** |
| Diabetes, no (%) |  |  |  |  | 0.414^c^ |
| No, no. (%) | 82(94.25) | 259(97.37) | 108(98.18) | 370(97.37) |  |
| Yes, no. (%) | 5(5.75) | 7(2.63) | 2(1.82) | 10(2.63) |  |
| Smoker |  |  |  |  | 0.918^c^ |
| No, no. (%) | 84(96.55) | 260(97.74) | 107(97.27) | 367(96.58) |  |
| Yes, no. (%) | 2(2.30) | 4(1.50) | 2(1.82) | 7(1.84) |  |
| Unknown, no. (%) | 1(1.15) | 2(0.75) | 1(0.91) | 6(1.58) |  |
| **Pre-operative patient-reported outcome data** | | | | | |
| BREAST-Q satisfaction with breast, mean (SD), 0-100 | 65.90(21.67) | 67.82(23.06) | 55.54(20.92) | 63.39(21.28) | **<0.001^b^** |
| BREAST-Q physical well-being chest and upper body, mean (SD), 0-100 | 94.53(6.75) | 91.16(9.48) | 63.28(10.14) | 75.44(9.03) | **<0.001^b^** |
| BREAST-Q psychosocial well-being, mean (SD), 0-100 | 76.25(17.05) | 76.18(16.89) | 63.23(15.31) | 70.51(17.15) | **<0.001^b^** |
| BREAST-Q physical well-being abdomen, mean (SD), 0-100 | 94.14(9.73) | 95.14(8.72) | 82.24(17.63) | 89.65(12.06) | **<0.001^b^** |
| BREAST-Q sexual well-being, mean (SD), 0-100 | 62.05(19.24) | 63.20(19.76) | 52.50(17.60) | 57.97(18.03) | **<0.001^b^** |
| **Follow-up patient-reported outcome data** | | | | | |
| BREAST-Q physical well-being chest and upper body at 3 months, mean (SD), 0-100 | 64.36(11.05) | 79.73(10.39) | 53.64(9.21) | 67.59(9.89) | **<0.001^b^** |
| BREAST-Q physical well-being chest and upper body at 12 months, mean (SD), 0-100 | 62.77(12.19) | 89.12(9.13) | 56.08(8.66) | 74.52(9.18) | **<0.001^b^** |
| BREAST-Q physical well-being chest and upper body at 24 months, mean (SD), 0-100 | 67.62(9.55) | 91.11(8.54) | 56.15(9.14) | 75.95(8.77) | **<0.001^b^** |
| BREAST-Q psychosocial well-being at 24 months, mean (SD), 0-100 | 72.25(17.90) | 82.62(17.55) | 59.14(15.94) | 72.68(18.39) | **<0.001^b^** |
| BREAST-Q sexual well-being at 24 months, mean (SD), 0-100 | 50.43(21.30) | 61.79(21.14) | 40.03(16.15) | 52.31(20.64) | **<0.001^b^** |
| BREAST-Q breast satisfaction at 24 months, mean (SD), 0-100 | 58.37(18.19) | 70.50(17.66) | 52.30(17.77) | 63.06(16.37) | **<0.001^b^** |
| Radiation |  |  |  |  | **<0.001^c^** |
| After reconstruction, no. (%) | 24 (27.59) | 27(10.15) | 36(32.73) | 54(14.21) |  |
| Before reconstruction, no. (%) | 4(4.60) | 16(6.02) | 2(1.82) | 14(3.68) |  |
| None, no. (%) | 59(67.82) | 223(83.83) | 72(65.45) | 312(82.11) |  |
| Mastectomy |  |  |  |  | **0.022^c^** |
| Nipple-sparing, no. (%) | 19(21.84) | 56(21.05) | 9(8.18) | 69(18.16) |  |
| Simple, no. (%) | 68(78.16) | 210(78.95) | 101(91.82) | 311(81.84) |  |
| Chemotherapy |  |  |  |  | **0.002^c^** |
| Received, no. (%) | 17(19.54) | 59(22.18) | 42(38.18) | 116(30.53) |  |
| Not received, no. (%) | 70(80.46) | 207(77.82) | 68(61.82) | 264(69.47) |  |
| Reconstruction laterality |  |  |  |  | 0.575^c^ |
| Unilateral, no. (%) | 38(43.68) | 101(37.97) | 38(34.55) | 139(36.58) |  |
| Bilateral, no. (%) | 49(56.32) | 165(62.03) | 72 (65.45) | 241(63.42) |  |
| Mastectomy indication |  |  |  |  | 0.113^c^ |
| Therapeutic, no. (%) | 74(85.06) | 227(85.34) | 102(92.73) | 341(89.74) |  |
| Prophylactic, no. (%) | 13(14.94) | 39(14.66) | 8(7.27) | 39(10.26) |  |
| Axillary intervention |  |  |  |  | **0.010^c^** |
| Axillary lymph node dissection (ALND), no. (%) | 28(32.18) | 55(20.68) | 43(39.09) | 115(30.26) |  |
| Sentinel lymph node biopsy (SLNB), no. (%) | 41(47.13) | 143(53.76) | 40(36.36) | 180(47.37) |  |
| None, no. (%) | 18(20.69) | 68(25.56) | 27(24.55) | 85(22.37) |  |
| **Socioeconomic and ethnic data** | | | | | |
| Marital status |  |  |  |  | 0.346^c^ |
| Partnerless, no. (%) | 13(14.94) | 39(14.66) | 23(20.91) | 71(18.68) |  |
| Partnered, no. (%) | 73(83.91) | 227(85.34) | 85(77.27) | 308(81.05) |  |
| Unknown, no. (%) | 1(1.15) |  | 2(1.82) | 1(0.26) |  |
| Education level |  |  |  |  | 0.332^c^ |
| High school degree and below, no. (%) | 2(2.30) | 15(5.64) | 8(7.27) | 28(7.37) |  |
| Above high school degree, no. (%) | 84(96.55) | 251(94.36) | 102(92.73) | 352(92.63) |  |
| Unknown, no. (%) | 1(1.15) |  |  |  |  |
| Working status |  |  |  |  | 0.779^c^ |
| Others, no. (%) | 25(28.74) | 76(28.57) | 37(33.64) | 110(28.95) |  |
| Employed, no. (%) | 60(68.97) | 187(70.30) | 72(65.45) | 268(70.53) |  |
| Unknown, no. (%) | 2(2.30) | 3(1.13) | 1(0.91) | 2(0.53) |  |
| Household income per year |  |  |  |  | **<0.001^c^** |
| <50,000$, no. (%) | 8(9.20) | 24(9.02) | 28(25.45) | 37(9.74) |  |
| $50,000 to $99,999$, no. (%) | 24(27.59) | 65(24.44) | 22(20.00) | 115(30.26) |  |
| >$100,000$, no. (%) | 52(59.77) | 169(63.53) | 57(51.82) | 216(56.84) |  |
| Unknown, no. (%) | 3(3.45) | 8(3.01) | 3(2.73) | 12(3.16) |  |
| Race |  |  |  |  | 0.247^c^ |
| White, no. (%) | 78(89.66) | 243(91.35) | 95(86.36) | 352(92.63) |  |
| Non-White, no. (%) | 8(9.20) | 21(7.89) | 14(12.73) | 26(6.84) |  |
| Unknown, no. (%) | 1(1.15) | 2(0.75) | 1(0.91) | 2(0.53) |  |

Note: *P* values < 0.05 highlighted in bold.

*^a^P* values refer to mean differences among the 4 classes of physical well-being trajectories.

*^b^P* values of one-way ANOVA test refer to the differences between the means of 4 classes of physical well-being trajectories.

*P* values of Chi-square test refer to the significance of the relationship between categorical variables.

**S1 Table 4. Baseline demographic and clinical characteristics of participants with autologous reconstruction in distinct physical well-being trajectories (n=581).**

|  | **Class1** | **Class2** | **Class 3** | **Class 4** | ***p*-value^a^** |
| --- | --- | --- | --- | --- | --- |
|  | medium high-restored  (n= 261) | low-not restored  (n= 28) | medium low-not restored  (n=187) | high-restored  (105) |  |
| **Patient variables** | | | | | |
| Age, mean (SD), years | 51.16(9.26) | 50.11(8.10) | 52.27(8.52) | 52.69(9.05) | 0.278^b^ |
| BMI, mean (SD), kg/m^2^ | 28.72(5.36) | 29.59(6.23) | 28.53(5.06) | 28.21(5.36) | 0.637^b^ |
| Diabetes, no (%) |  |  |  |  | 0.588^c^ |
| No, no. (%) | 247(94.64) | 26(92.86) | 171(91.44) | 98(93.33) |  |
| Yes, no. (%) | 14(5.36) | 2(7.14) | 16(8.56) | 7(6.67) |  |
| Smoker |  |  |  |  | **0.042^c^** |
| No, no. (%) | 257(98.47) | 26(92.86) | 181(96.79) | 105(100) |  |
| Yes, no. (%) | 3(1.15) | 2(7.14) | 5(2.67) | 0(0) |  |
| Unknown, no. (%) | 1(0.38) |  | 1(0.53) |  |  |
| **Pre-operative patient-reported outcome data** | | | | | |
| BREAST-Q satisfaction with breast, mean (SD), 0-100 | 55.56(19.99) | 49.57(20.54) | 50.79(21.64) | 63.70(20.29) | **<0.001^b^** |
| BREAST-Q physical well-being chest and upper body, mean (SD), 0-100 | 79.20(11.02) | 56.93(14.54) | 67.17(11.77) | 92.90(9.04) | **<0.001^b^** |
| BREAST-Q psychosocial well-being, mean (SD), 0-100 | 66.52(16.88) | 54.43(17.43) | 63.10(19.23) | 74.73(18.08) | **<0.001^b^** |
| BREAST-Q physical well-being abdomen, mean (SD), 0-100 | 87.95(13.85) | 73.71(18.41) | 84.96(14.28) | 95.58(7.88) | **<0.001^b^** |
| BREAST-Q sexual well-being, mean (SD), 0-100 | 50.43(19.92) | 47.50(23.99) | 46.88(21.23) | 56.65(22.28) | **0.002^b^** |
| **Follow-up patient-reported outcome data** | | | | | |
| BREAST-Q physical well-being chest and upper body at 3 months, mean (SD), 0-100 | 75.25(10.03) | 49.96(9.23) | 61.43(9.51) | 86.39(10.76) | **<0.001^b^** |
| BREAST-Q physical well-being chest and upper body at 12 months, mean (SD), 0-100 | 78.04(9.31) | 46.04(9.24) | 63.72(7.98) | 94.31(7.60) | **<0.001^b^** |
| BREAST-Q physical well-being chest and upper body at 24 months, mean (SD), 0-100 | 79.82(9.15) | 41.93(11.24) | 63.50(8.82) | 93.14(8.66) | **<0.001^b^** |
| BREAST-Q psychosocial well-being at 24 months, mean (SD), 0-100 | 76.22(17.72) | 57.25(16.63) | 70.57(18.94) | 83.46(18.16) | **<0.001^b^** |
| BREAST-Q sexual well-being at 24 months, mean (SD), 0-100 | 56.50(21.06) | 44.21(24.27) | 51.67(22.28) | 65.19(23.66) | **<0.001^b^** |
| BREAST-Q breast satisfaction at 24 months, mean (SD), 0-100 | 68.50(18.60) | 56.00(17.53) | 63.07(18.68) | 75.25(17.89) | **<0.001^b^** |
| Radiation |  |  |  |  | **0.049^c^** |
| After reconstruction, no. (%) | 54(20.69) | 10(35.71) | 54(28.88) | 15(14.29) |  |
| Before reconstruction, no. (%) | 67(25.67) | 5(17.86) | 42(22.46) | 24(22.86) |  |
| None, no. (%) | 140(53.64) | 13(46.43) | 91(48.66) | 66(62.86) |  |
| Mastectomy |  |  |  |  | 0.960^c^ |
| Nipple-sparing, no. (%) | 5(1.92) | 0(0) | 5(2.67) | 2(1.9) |  |
| Simple, no. (%) | 256(98.08) | 28(100) | 182(97.33) | 103(98.1) |  |
| Chemotherapy |  |  |  |  | **0.021^c^** |
| Received, no. (%) | 75(28.74) | 8(28.57) | 72(38.5) | 23(21.9) |  |
| Not received, no. (%) | 186(71.26) | 20(71.43) | 115(61.5) | 82(78.1) |  |
| Reconstruction laterality |  |  |  |  | 0.509^c^ |
| Unilateral, no. (%) | 144(55.17) | 16(57.14) | 109(58.29) | 67(63.81) |  |
| Bilateral, no. (%) | 117(44.83) | 12(42.86) | 78(41.71) | 38(36.19) |  |
| Mastectomy indication |  |  |  |  | 0.135^c^ |
| Therapeutic, no. (%) | 234(89.66) | 27(96.43) | 177(94.65) | 100(95.24) |  |
| Prophylactic, no. (%) | 27(10.34) | 1(3.57) | 10(5.35) | 5(4.76) |  |
| Axillary intervention |  |  |  |  | 0.505^c^ |
| Axillary lymph node dissection (ALND), no. (%) | 58(22.22) | 11(39.29) | 45(24.06) | 24(22.86) |  |
| Sentinel lymph node biopsy (SLNB), no. (%) | 106(40.61) | 9(32.14) | 77(41.18) | 48(45.71) |  |
| None, no. (%) | 97(37.16) | 8(28.57) | 65(34.76) | 33(31.43) |  |
| **Socioeconomic and ethnic data** | | | | | |
| Marital status |  |  |  |  | 0.214^c^ |
| Partnerless, no. (%) | 44(16.86) | 9(32.14) | 30(16.04) | 16(15.24) |  |
| Partnered, no. (%) | 217(83.14) | 19(67.86) | 155(82.89) | 89(84.76) |  |
| Unknown, no. (%) |  |  | 2(1.07) |  |  |
| Education level |  |  |  |  | 0.216^c^ |
| High school degree and below, no. (%) | 37(14.18) | 7(25) | 22(11.76) | 11(10.48) |  |
| Above high school degree, no. (%) | 223(85.44) | 21(75) | 165(88.24) | 94(89.52) |  |
| Unknown, no. (%) | 1(0.38) |  |  |  |  |
| Working status |  |  |  |  | 0.195^c^ |
| Others, no. (%) | 70(26.82) | 12(42.86) | 45(24.06) | 32(30.48) |  |
| Employed, no. (%) | 187(71.65) | 16(57.14) | 140(74.87) | 73(69.52) |  |
| Unknown, no. (%) | 4(1.53) |  | 2(1.07) |  |  |
| Household income per year |  |  |  |  | **0.049^c^** |
| <50,000$, no. (%) | 46(17.62) | 10(35.71) | 39(20.86) | 19(18.10) |  |
| $50,000 to $99,999$, no. (%) | 101(38.70) | 10(35.71) | 86(45.99) | 37(35.24) |  |
| >$100,000$, no. (%) | 103(39.46) | 7(25.00) | 57(30.48) | 48(45.71) |  |
| Unknown, no. (%) |  | 1(3.57) | 5(2.67) | 1(0.95) |  |
| Race |  |  |  |  | **0.022^c^** |
| White, no. (%) | 237(90.80) | 23(82.14) | 162(86.63) | 1 00(95.24) |  |
| Non-White, no. (%) | 22(8.43) | 5(17.86) | 24(12.83) | 4(3.81) |  |
| Unknown, no. (%) | 2(0.77) |  | 1(0.53) | 1(0.95) |  |

Note: *P* values < 0.05 highlighted in bold.

^a^*P* values refer to mean differences among the 4 classes of physical well-being trajectories.

*^b^P* values of one -way ANOVA test refer to the differences between the means of 4 classes of physical well-being trajectories.

*^c^P* values of Chi-square test refer to the significance of the relationship between categorical variables.

**S1 Table 5. Multinomial logistic regression models predicting breast satisfaction outcome based on class membership of physical well-being trajectory**

|  | **Implant-based reconstruction**  **(n=793）** | |  | **Autologous reconstruction**  **(n=549)** | |
| --- | --- | --- | --- | --- | --- |
|  | Improved vs stable^b^ | Worsened vs stable^b^ |  | Improved vs stable^b^ | Worsened vs stable^b^ |
| Class^a^ |  |  |  |  |  |
| high-restored | 2.36 | 0.68 |  | 3.21 | 1.23 |
| medium high-not restored | 1.97 | 0.95 |  |  |  |
| medium low-restored | 2.60* | 1.04 |  |  |  |
| medium high-restored |  |  |  | 3.67 | 2.32 |
| medium low- not restored |  |  |  | 3.20 | 2.14 |
| Laterality: unilateral | 0.79 | 1.27 |  | 1.29 | 1.42 |
| Indication: therapeutic | 1.98 | 3.42* |  | 0.51 | 0.87 |
| Mastectomy: simple | 0.95 | 1.02 |  | 4.17 | 0.94 |
| Axillary |  |  |  |  |  |
| none | 2.46 | 2.23 |  | 1.32 | 1.24 |
| SLNB | 1.90* | 1.66 |  | 1.32 | 1.01 |
| BMI | 1.00 | 0.96 |  | 1.00 | 0.96 |
| Diabetes: no | 0.00*** | 0.00*** |  | 0.66 | 0.39 |
| Radiation |  |  |  |  |  |
| before | 0.29 | 0.14** |  | 3.01 | 1.15 |
| none | 0.34* | 0.25** |  | 1.30 | 0.98 |
| Chemotherapy: yes | 0.61 | 0.81 |  | 0.75 | 1.25 |
| Age | 1.00 | 0.99 |  | 0.96 | 0.95* |
| Smoker: no | 0.84 | 0.73 |  | 0.00*** | 0.00*** |
| Marital: partnerless | 0.68 | 1.23 |  | 0.79 | 1.07 |
| Education: High school and below | 0.67 | 0.56 |  | 0.61 | 0.89 |
| Work: others | 0.83 | 1.14 |  | 1.06 | 0.83 |
| Income |  |  |  |  |  |
| $50,000-$99,999 | 2.51** | 2.28* |  | 1.83 | 1.56 |
| Less than $50,000 | 1.64 | 1.21 |  | 1.31 | 0.83 |
| Race: white | 0.27* | 0.47 |  | 0.32 | 0.40 |
| Baseline PRO psychosocial | 0.97** | 1.01 |  | 0.97** | 1.02 |
| Baseline PRO physical abdomen | 0.98 | 1.00 |  | 0.99 | 1.00 |
| Baseline PRO sexual | 0.98 | 1.00 |  | 1.00 | 1.02 |

^a^The reference group for class membership is “low-not restored”. The independent variables were all measured at baseline. Coefficients are relative risk ratio. Risk ratios were approaching 0 for some variables due to the small sample size for that variable.

^b^Increase or decrease at least by minimal clinically important difference compared to baseline (4 for breast satisfaction in this study). Standard errors are robust. **p*<0.05; ***p*<0.01; ****p*<0.001.

**S1 Table 6. Multinomial logistic regression models predicting sexual well-being outcome based on class membership of physical well-being trajectory.**

|  | **Implant-based reconstruction**  **(n=793）** | |  | **Autologous(flap) reconstruction**  **(n=549)** | |
| --- | --- | --- | --- | --- | --- |
|  | Improved vs stable^b^ | Worsened vs stable^b^ |  | Improved vs stable^b^ | Worsened vs stable^b^ |
| Class^a^ |  |  |  |  |  |
| high-restored | 1.30 | 0.35* |  | 1.07 | 0.14* |
| medium high-not restored | 1.08 | 0.47 |  |  |  |
| medium low-restored | 1.36 | 0.56 |  |  |  |
| medium high-restored |  |  |  | 1.02 | 0.21 |
| medium low- not restored |  |  |  | 1.34 | 0.44 |
| Laterality: unilateral | 1.22 | 1.14 |  | 1.03 | 0.60 |
| Indication: therapeutic | 0.66 | 0.51 |  | 0.76 | 0.34 |
| Mastectomy: simple | 0.58 | 0.93 |  | 10.10* | 1.95 |
| Axillary |  |  |  |  |  |
| none | 0.73 | 0.54 |  | 0.97 | 0.31* |
| SLNB | 0.67 | 0.62 |  | 0.99 | 0.53 |
| BMI | 1.00 | 1.03 |  | 0.97 | 0.92** |
| Diabetes: no | 0.00*** | 0.00*** |  | 1.85 | 0.34 |
| Radiation |  |  |  |  |  |
| before | 2.13 | 0.99 |  | 1.70 | 1.37 |
| none | 2.03 | 1.32 |  | 1.81 | 1.84 |
| Chemotherapy: yes | 1.30 | 2.23** |  | 0.68 | 1.01 |
| Age | 1.01 | 0.99 |  | 1.03 | 1.02 |
| Smoker: no | 2.59 | 8.43** |  | 3.98 | 0.46 |
| Marital: partnerless | 1.33 | 1.92* |  | 1.09 | 2.62* |
| Education: High school and below | 2.27 | 1.35 |  | 1.36 | 0.96 |
| Work: others | 1.06 | 1.53 |  | 1.27 | 1.23 |
| Income |  |  |  |  |  |
| $50,000-$99,999 | 1.07 | 0.75 |  | 0.95 | 0.92 |
| Less than $50,000 | 1.69 | 1.23 |  | 2.94* | 1.16 |
| Race: white | 0.77 | 1.37 |  | 0.49 | 0.63 |
| Baseline PRO psychosocial | 0.99 | 1.01 |  | 1 | 1 |
| Baseline PRO physical abdomen | 1 | 1 |  | 0.99 | 1.01 |
| Baseline PRO breast | 0.99 | 1.01* |  | 0.98* | 1.02 |

^a^The reference group for class membership is “low-not restored”. The independent variables were all measured at baseline. Coefficients are relative risk ratio. Risk ratios were approaching 0 for some variables due to the small sample size for that variable.

^b^Increase or decrease at least by minimal clinically important difference compared to baseline (4 for sexual well-beings in this study). Standard errors are robust. **p*<0.05; ***p*<0.01; ****p*<0.001.

**S1 Table 7. Multinomial logistic regression models predicting psychosocial well-being outcome based on class membership of physical well-being trajectory.**

|  | **Implant-based reconstruction**  **(n=793）** | |  | **Autologous reconstruction**  **(n=549)** | |
| --- | --- | --- | --- | --- | --- |
|  | Improved vs stable^b^ | Worsened vs stable^b^ |  | Improved vs stable^b^ | Worsened vs stable^b^ |
| Class^a^ |  |  |  |  |  |
| high-restored | 1.25 | 0.32** |  | 1.27 | 0.62 |
| medium high-not restored | 1.39 | 0.86 |  |  |  |
| medium low-restored | 1.00 | 0.51 |  |  |  |
| medium high-restored |  |  |  | 1.55 | 0.68 |
| medium low- not restored |  |  |  | 1.64 | 1.48 |
| Laterality: unilateral | 0.96 | 1.02 |  | 0.90 | 0.65 |
| Indication: therapeutic | 1.46 | 1.92 |  | 1.73 | 0.97 |
| Mastectomy: simple | 0.98 | 1.44 |  | 12.14** | 2.23 |
| Axillary |  |  |  |  |  |
| none | 1.03 | 0.86 |  | 0.88 | 0.33* |
| SLNB | 1.53 | 1.44 |  | 0.87 | 0.54 |
| BMI | 0.99 | 1.01 |  | 1.00 | 1.06 |
| Diabetes: no | 0.28 | 0.60 |  | 0.49 | 0.84 |
| Radiation |  |  |  |  |  |
| before | 1.60 | 1.47 |  | 0.78 | 1.16 |
| none | 0.75 | 0.55 |  | 1.10 | 1.34 |
| Chemotherapy: yes | 0.86 | 1.19 |  | 0.66 | 0.69 |
| Age | 1 | 1 |  | 1.02 | 0.99 |
| Smoker: no | 0.45 | 0.83 |  | 13.60** | 2.93 |
| Marital: partnerless | 0.54* | 0.87 |  | 0.41** | 0.70 |
| Education: High school and below | 0.99 | 0.53 |  | 0.94 | 1.14 |
| Work: others | 0.80 | 1.03 |  | 0.89 | 0.76 |
| Income |  |  |  |  |  |
| $50,000-$99,999 | 0.97 | 0.84 |  | 1.35 | 1.20 |
| Less than $50,000 | 1.03 | 1.33 |  | 1.86 | 1.36 |
| Race: white | 0.77 | 1.13 |  | 0.70 | 1.05 |
| Baseline PRO breast | 0.99** | 1.01 |  | 0.97*** | 1.00 |
| Baseline PRO physical abdomen | 0.98 | 0.99 |  | 0.99 | 0.99 |
| Baseline PRO sexual | 0.99 | 1.01 |  | 0.98** | 0.99 |

^a^The reference group for class membership is “low-not restored”. The independent variables were all measured at baseline. Coefficients are relative risk ratio.

^b^Increase or decrease at least by minimal clinically important difference compared to baseline (4 for psychosocial well-beings in this study). Standard errors are robust. **p*<0.05; ***p*<0.01; ****p*<0.001.
