# Supplementary material for: Physical well-being recovery trajectories by reconstruction modality in women undergoing mastectomy and breast reconstruction: Significant predictors and health-related quality of life outcomes
Source: PLoS One. 2023 Jul 28;18(7):e0289182. doi: 10.1371/journal.pone.0289182 (PMC10381031; doi:10.1371/journal.pone.0289182)
Supplement: Attachment — Submitted filename: Response to Reviewers.docx [file pone.0289182.s003.docx]

**Cover letter**

**Dear editors and reviewers,**

We are grateful for the effort expended in reviewing our manuscript entitled “Physical well-being recovery trajectories by reconstruction modality in women undergoing mastectomy and breast reconstruction: significant predictors and health-related quality of life outcomes.” We are delighted to have received high quality reviews which we were able to respond to positively.

**Role of Funder statement**

This study is supported by National Cancer Institute Grant No. R01 CA152192 and in part by National Cancer Institute Support Grant No. P30 CA008748, but the funders had no role in study design, data collection and analysis, decision to publish, or preparation of the manuscript.

**Competing Interests statement**

Our competing interests statement does not alter our adherence to PLOS ONE policies on sharing data and materials.

We include the amended manuscript alongside point-by-point commentary herewith.

Kind regards,

Cai

**Responses to Reviewer1**

**Reviewer #1:** This paper describes the longitudinal changes in physical well-being of the chest up to 2 years after breast reconstruction by surgical technique using latent class growth analysis (LCGA) and identify factors influencing this change.

The statistical analysis is well described, and the results are clearly presented.

The main research findings of this paper will be important for understanding of quality of life after breast reconstruction, contributing to improved patient care.

I would recommend it for acceptance after the minor points listed below.

**Response:**

Thank you for your valuable feedback and positive assessment of our paper. We appreciate your recognition of the clarity in our statistical analysis and presentation of results, as well as the significance of our research findings for enhancing quality of life after breast reconstruction and improving patient care.

Your detailed suggestions have greatly contributed to refining and strengthening our paper. We are fully committed to upholding the highest research standards and greatly value your expertise in helping us achieve this goal.

We have incorporated the corresponding changes into the manuscript's main body, highlighted in red, and provided a detailed explanation for each change below.

1)Regarding Table 1, there are differences in the four PWBC trajectories between IMPG and AUTOG (e.g., IMPG includes 'medium high-not restored' but AUTOG does not). To facilitate the reader's visual understanding, it is suggested that this be corrected so that the class and graph color corresponds one-to-one.

**Response:**

Thanks for this good suggestion.

We have adjusted the colors in both graphs to establish a one-to-one correspondence between the classes and the graphs, to facilitate the reader's visual understanding. See these new plots below.

2)The second line of the abstract states "in patients who underwent postmastectomy and breast reconstruction,". Would it be better to correct it to "postmastectomy breast reconstruction" or "mastectomy and breast reconstruction"?

**Response:**

Thanks for pointing out these details.

I took your advice and corrected this sentence to read as “in patients who underwent postmastectomy ~~and~~ breast reconstruction” in Abstract.

Actually in main body of context, it keeps using “postmastectomy breast reconstruction”, or PMBR for short.

PMBR=postmastectomy breast reconstruction

3)"his/" is inappropriate because the study subjects are all women. (p.10; line10)

**Response**:

We agree with the reviewer’ comments and thanks for this interesting suggestion.

We removed the word” his/”, our patients here are all women. See page 10 for details.

*“each included patient will be assigned a new class membership based on their respective posterior class probabilities to represent ~~his/~~her growth patterns over time, and their respective new class membership attribution will not change at all the assessment time points.”*

4)The grammar of "The physical well-being score was AUTOG is significantly better than that in IMPG[11,28]." should be corrected. (p.21; lines18-19)

**Response:** Thanks for your helpful suggestions.

I took you advice and changed this sentence in p.21 to this:

*“The physical well-being score in AUTOG is significantly better than that in IMPG[11,28].”*

**Responses to Journal requirements:**

When submitting your revision, we need you to address these additional requirements.

1. Please ensure that your manuscript meets PLOS ONE's style requirements, including those for file naming. The PLOS ONE style templates can be found at

<https://journals.plos.org/plosone/s/file?id=wjVg/PLOSOne_formatting_sample_main_body.pdf> and

<https://journals.plos.org/plosone/s/file?id=ba62/PLOSOne_formatting_sample_title_authors_affiliations.pdf>

**Response:** Yes. Our manuscript meets PLOS ONE's style requirements.

2. Please include a complete copy of PLOS’ questionnaire on inclusivity in global research in your revised manuscript. Our policy for research in this area aims to improve transparency in the reporting of research performed outside of researchers’ own country or community. The policy applies to researchers who have travelled to a different country to conduct research, research with Indigenous populations or their lands, and research on cultural artefacts. The questionnaire can also be requested at the journal’s discretion for any other submissions, even if these conditions are not met.  Please find more information on the policy and a link to download a blank copy of the questionnaire here: <https://journals.plos.org/plosone/s/best-practices-in-research-reporting>. Please upload a completed version of your questionnaire as Supporting Information when you resubmit your manuscript.

**Response:** Yes, we added a subsection ‘Inclusivity in global research’ to our Methods section and adding the following sentence: “Additional information regarding the ethical, cultural, and scientific considerations specific to inclusivity in global research is included in the Supporting Information (S2 Checklist)” in p.10.

Of note, the study was originally IRB approved in 2012 which is before single IRB requirements. Each individual center managed its own regulatory process with support from us.

We completed this questionnaire and uploaded it as Supporting Information when we resubmit our manuscript.

3. Thank you for stating the following financial disclosure:

“Supported by National Cancer Institute Grant No. R01 CA152192 and in part by National Cancer Institute Support Grant No. P30 CA008748.”

Please state what role the funders took in the study.  If the funders had no role, please state: "The funders had no role in study design, data collection and analysis, decision to publish, or preparation of the manuscript."

If this statement is not correct you must amend it as needed.

Please include this amended Role of Funder statement in your cover letter; we will change the online submission form on your behalf.

**Response:**  **Role of Funder statement**

The funders had no role in study design, data collection and analysis, decision to publish, or preparation of the manuscript.

4. Thank you for stating the following in the Competing Interests section:

“Cai Xu

No relationship to disclose.

Peiyi Lu

No relationship to disclose.

André Pfob

No relationship to disclose.

Andrea L. Pusic

Patents, Royalties, Other Intellectual Property: I am a codeveloper of BREAST-Q and receive royalty payments when it is used in for-profit industry-sponsored trials.

Jennifer B Hamill

No relationship to disclose.

Chris Sidey-Gibbons

No relationship to disclose.”

Please confirm that this does not alter your adherence to all PLOS ONE policies on sharing data and materials, by including the following statement: "This does not alter our adherence to PLOS ONE policies on sharing data and materials.” (as detailed online in our guide for authors <http://journals.plos.org/plosone/s/competing-interests>).  If there are restrictions on sharing of data and/or materials, please state these. Please note that we cannot proceed with consideration of your article until this information has been declared.

Please include your updated Competing Interests statement in your cover letter; we will change the online submission form on your behalf.

**Response:** **Competing Interests statement**

This does not alter our adherence to PLOS ONE policies on sharing data and materials.

5. Please review your reference list to ensure that it is complete and correct. If you have cited papers that have been retracted, please include the rationale for doing so in the manuscript text, or remove these references and replace them with relevant current references. Any changes to the reference list should be mentioned in the rebuttal letter that accompanies your revised manuscript. If you need to cite a retracted article, indicate the article’s retracted status in the References list and also include a citation and full reference for the retraction notice.

**Response:**

All references listed in this manuscript are complete and correct. No new references were added during the revision process.
